# Epigenetic and Transcriptomic Alterations of Protein Aggregation-Linked Genes in Suicide: A Pilot Study

**DOI:** 10.3390/genes16121467

**Published:** 2025-12-08

**Authors:** Taja Bedene, Julija Šmon, Alja Videtič Paska, Tomaž Zupanc, Katarina Kouter

**Affiliations:** 1 Faculty of Medicine, University of Ljubljana, 1000 Ljubljana, Slovenia; taja.bedene10@gmail.com; 2 Institute of Biochemistry and Molecular Genetics, Faculty of Medicine, University of Ljubljana, 1000 Ljubljana, Slovenia; julija.smon@mf.uni-lj.si (J.Š.); alja.videtic@mf.uni-lj.si (A.V.P.); 3 Institute of Forensic Medicine, Faculty of Medicine, University of Ljubljana, 1000 Ljubljana, Slovenia; tomaz.zupanc@mf.uni-lj.si; 4 Institute of Microbiology and Immunology, Faculty of Medicine, University of Ljubljana, 1000 Ljubljana, Slovenia

**Keywords:** suicidality, psychiatry, epigenetics, proteostasis, protein aggregation

## Abstract

**Background/Objectives**: Suicide is a significant public health concern with a multifactorial etiology. The biological background of suicide is not sufficiently explored, which encumbers suicide prevention. Epigenetic mechanisms may mediate environmental influences on suicide risk. Recent studies have suggested that protein aggregation occurs in the brains of patients with chronic psychiatric disorders and suicidality, which may influence disease trajectory. However, the intersection between epigenetics and proteinopathy in suicide remains unexplored. Our pilot study investigated whether aggregation-related genes show epigenetic and transcriptional alterations in the post-mortem brains of individuals who had died by suicide. **Methods**: Brain tissue from 69 male subjects (32 suicide by hanging, 37 sudden cardiac death controls) was collected at autopsy. Genome-wide hippocampal DNA methylation data from our previous reduced representation bisulfite sequencing (RRBS) study were reanalyzed to identify differentially methylated cytosines (DMCs) in candidate aggregation-related genes. The expression of nine candidate and three reference genes in the hippocampus and Brodmann area 46 was assessed by qPCR. Statistical analyses were performed using Student’s *t*-test or Mann–Whitney U test (*p* < 0.05 was considered significant). **Results:** Reanalysis revealed hypomethylation in suicide cases within *CRMP1*, *DISC1*, *MAPT*, *SOD1*, *PRKN*, *GABARAPL1*, *GRIN2A,* and *GRIN2B*. In the hippocampus, suicides exhibited increased expression of *CRMP1*, *SOD1*, *PRKN*, *GABARAPL1*, and *GRIN2A*, and decreased *MAPT* expression. The *GRIN2A/GRIN2B* ratio was significantly elevated. In Brodmann area 46, altered expression was limited to increased *GRIN2A* and decreased *DISC1*. **Conclusions**: This is the first study to implicate epigenetic and transcriptional dysregulation of protein aggregation-associated genes in suicide. The findings suggest a possible role for proteostasis disturbances in suicidality, particularly within the hippocampal pathways related to stress response and synaptic signaling. Validation in larger cohorts and protein-level studies are warranted to determine the functional significance of these findings.

## 1. Introduction

Every year, suicide accounts for over 700,000 deaths globally and is the fourth leading cause of death among young adults [1]. Suicide is defined as an intentional self-directed act that results in death [2], while suicidal behavior also includes non-lethal attempts, which occur even more often [3]. Suicide affects all age groups, nationalities, and both sexes; however, men have 2.3-times higher suicide rates than women [1]. Suicide is often, but not always, accompanied by comorbid chronic psychiatric disorders, most notably major depressive disorder (MDD) [4] and substance use disorder [2]. Efforts at suicide prevention are currently insufficient [5], and the discovery of reliable biomarkers for suicide risk prediction is of high priority [6].

Epidemiological studies have estimated the heritability of suicide risk at 40% [7], suggesting that biological parameters may serve as biomarkers for suicide risk. This was further supported by genetic research, which began with a candidate gene-driven approach and has since expanded towards non-candidate driven approaches, enabled by high- throughput sequencing technologies [8]. However, as individual loci had small effect sizes, genome-wide association studies (GWAS) did not produce clinically applicable findings [9]. Multi-omics studies have linked various biological processes to suicidality, most notably (neuro) inflammation, altered neurotransmission, synaptic dysfunction, and the dysregulation of the hypothalamic–pituitary–adrenal (HPA) axis [10,11]. However, the etiology of suicidal behavior is complex, being influenced by biological, psychological, socio-economic, and environmental risk factors [3,12]. As described by the stress–diathesis model, risk factors act distally and/or proximally to promote suicidal behavior [13]. In this regard, epigenetic modifications are of particular interest as they may mediate the long-term effects of environmental stressors, such as childhood trauma, on the brain [14]. The term “epigenetics” refers to heritable chemical DNA alterations that are responsive to environmental triggers and influence gene activity without altering the nucleotide sequence; examples include DNA methylation, post-translational histone modifications, and regulation by non-coding RNAs [15]. This mediating role of epigenetic mechanisms is supported by animal studies [16,17] as well as studies of individuals with a history of childhood trauma who had died by suicide, which associated decreased glucocorticoid receptor gene expression in the hippocampus to hypermethylation of its promoter [18]. Thus, epigenetic modifications represent a plausible molecular link between environmental risk factors and biological changes observed in suicidality, making them attractive targets for biomarker and mechanistic studies. The most characterized epigenetic modification is DNA methylation, and human suicidality studies conducted on the post-mortem brain have reported differentially methylated loci potentially associated with suicide in various brain regions [19,20,21].

Recently, protein aggregation has been proposed as a potential pathophysiological process contributing to the development of psychiatric disorders [22,23,24]. Protein aggregates have been thoroughly examined in a variety of neurodegenerative disorders, including Alzheimer’s disease (AD), Parkinson’s disease (PD), frontotemporal dementia (FTD), and amyotrophic lateral sclerosis (ALS) [25]. Approximately 65% of patients with a neurodegenerative disorder also present with psychiatric symptoms, such as disturbances in affect and behavior [26,27]. Furthermore, comorbid psychiatric disorders, especially MDD and mood disorders, are prevalent among these patients [28]. On the other hand, individuals diagnosed with psychiatric disorders have an up to four times increased risk of subsequently developing a neurodegenerative disorder [27]. Recently, it has been suggested that specific protein aggregates (or insoluble protein species) may also be present in the brains of individuals with chronic psychopathologies, including schizophrenia [29] and MDD, as well as those who had died by suicide [30]. Studies have mostly focused on protein insolubility and ubiquitination as markers of protein aggregation. However, a recent publication on olfactory neuronal cells, derived from the nasal biopsies of living schizophrenia patients and healthy controls, successfully visualized ubiquitin-positive protein aggregates in a subset of patient cells [31]. Still, protein aggregation in suicidal behavior has not yet been systematically studied, and whether it contributes to suicide pathophysiology, and through what mechanisms, remains unknown. Genes coding for potentially aggregating proteins in mental disorders are mostly distinct from those occurring in neurodegenerative disorders and include *CRMP1* (collapsin response mediator protein 1), *DISC1* (disrupted-in-schizophrenia 1), *DTNBP1* (dystrobrevin-binding protein 1), *NPAS3* (neuronal PAS domain protein 3), *TRIOBP* (TRIO and F-actin binding protein), and possibly others [32]. Several of the aggregation-related genes analyzed in this study overlap with the loci previously implicated in psychiatric disorders and suicidality. For example, *DISC1* has long been recognized as a genetic risk factor for schizophrenia and affective disorders [33], and GWAS have linked the loci associated with the *DISC1* interactome to suicidality [34]. *GRIN2B* variants have been associated with treatment-resistant depression [35]. *MAPT* has also been linked to mood dysregulation and cognitive symptoms in psychiatric populations.

The role of aggregates in disease progression is not clear; while in neurodegenerative disorders, protein aggregation is linked to neurotoxicity and neuronal death [36], this is not the case in mental disorders, where it may disrupt neuronal function more subtly [23]. However, in both types of brain disorder, aggregates are speculated to contribute to processes such as oxidative stress and neuroinflammation [22]. Compared to aggregates in neurodegenerative disorders, those occurring in psychiatric disorders seem to be detectable only at the submicroscopic level [33]. Furthermore, it is not yet clear to what extent these psychopathology-specific proteins aggregate together or independently of each other in patient subgroups. Currently, *DISC1*, *DTNBP1*, and *NPAS3* have been shown to promote the aggregation of additional proteins through co-aggregation, while some aggregates demonstrate the ability to spread between cells in in vitro studies [30].

Protein aggregation is a dynamic process that likely occurs as a consequence of genetic and environmental influences. Epigenetic dysregulation has been described in neurodegenerative disorders [37]; for example, there is evidence that DNA methylation of the genes related to the Tau protein function in the brain is involved in AD pathogenesis [38]. Studies examining proteostasis in psychiatric disorders have demonstrated that upregulation of specific genes increases the tendency of these proteins to aggregate [22,39]. Gene expression is regulated at multiple levels, one of which is epigenetic regulation [21]. Therefore, it is important to consider possible epigenetic alterations of aggregation-prone proteins in psychopathologies. As altered mRNA levels may be present in earlier disease stages, when proteostasis is not yet significantly disturbed, mRNA expression studies may assess the influence of epigenetic modifications on gene expression and provide insights into molecular disturbances that precede protein-level alterations.

Despite recent evidence linking protein aggregation to psychiatric disorders and the growing number of studies examining epigenetic mechanisms in suicide, an important knowledge gap remains. While DNA methylation of aggregation-related genes has been studied in neurodegenerative disorders, the intersection between epigenetics and proteostasis in suicidal behavior has not yet been investigated. Furthermore, the molecular mechanisms driving aggregation-prone protein expression in psychiatric disorders remain unknown. This is significant because epigenetic modifications may represent early, potentially reversible changes that precede proteostasis disturbance and offer a window of opportunity for early intervention. Additionally, the functional significance of possible epigenetic changes in aggregation-related genes has not yet been assessed. As gene expression is regulated by epigenetic mechanisms, it is plausible that epigenetic modifications of aggregation-prone genes contribute to molecular disturbances observed in psychiatric disorders, and transcriptional analyses may provide insights into early molecular events in these processes.

We therefore hypothesize that suicide is associated with altered DNA methylation and transcriptional regulation of the genes involved in protein aggregation and proteostasis. To address this, we investigated epigenetic and transcriptional alterations of candidate genes coding for aggregation-prone proteins in post-mortem brain tissue from individuals who had died by suicide. Specifically, we reanalyzed results from our recent exploratory genome-wide study examining methylation patterns in the hippocampi of individuals who had died by suicide, focusing on altered methylation patterns in the genes associated with disrupted proteostasis in psychiatric or neurodegenerative disorders [21]. Furthermore, we complemented the analysis with selected gene expression studies on the subjects’ hippocampi and Brodmann area 46 (BA46) of the prefrontal cortex to evaluate the functional significance of these altered methylation patterns.

## 2. Materials and Methods

### 2.1. Subjects

Altogether, 69 male subjects were included in this study. Cause of death was suicide by hanging (32 subjects, case group) or sudden cardiac death (37 subjects, control group). The subjects were residents of the Republic of Slovenia and were aged between 18 and 65 years. Brain tissue samples were collected during routine autopsy and snap frozen in liquid nitrogen. Toxicology and alcoholimetric testing were performed as a part of routine procedures. Tissue was then stored at −80 °C until further processing. The characteristics of the subjects are listed in Table 1 (additional information is listed in Supplementary Table S1). When comparing the age of the subjects, differences can be observed. Namely, the subjects of the control group are older than the subjects in the case group. This is probably due to the control group cause-of-death selection, as sudden cardiac death is less common in the younger population. The post-mortem interval did not differ statistically significantly between the two groups. All tissue samples were collected by the same forensic medical examiner to ensure the standardization of the procedure.

Exclusion criteria were age, sex, insufficient body preservation, and cause of death. We excluded individuals older than 65 years due to the possible presence of age-related neurodegeneration, women due to the marked predominance of suicides among men, and individuals whose method of suicide was not hanging, as this ensured greater homogeneity of the group. We also excluded individuals with tissue damage associated with alcohol abuse due to the metabolic and epigenetic effects of alcohol on the brain. This study was approved by the Slovenian National Medical Ethics Committee.

### 2.2. Brain Region Selection

We selected two brain regions for inclusion in this study: the hippocampus and BA46. Both have already been implicated in suicidality [40]. The hippocampus, part of the limbic system, is strongly involved in emotion, various behaviors, motivation, and memory [41]. It is located in the medial temporal lobe of the brain and plays a key role in the formation, organization, and storage of long-term memory [42]. It receives information through different types of neurons, including serotonergic, noradrenergic, GABAergic, and dopaminergic neurons [43]. The limbic system is functionally connected to the prefrontal cortex, which contains Brodmann areas 9 and 46. BA46 is located in the frontal cortex, specifically in the dorsolateral prefrontal cortex. The dorsolateral prefrontal cortex is an evolutionarily very recent development and is involved in maintaining attention, working memory, planning, decision-making, and self-control [44,45,46].

### 2.3. DNA Methylation Reanalysis and Gene Selection

A previous pilot genome-wide methylation study, which we conducted and published in 2019 [21], provided a large amount of data. In order to identify a set of candidate genes involving protein aggregation in suicidality, we re-examined the previously obtained data on DNA methylation in the hippocampus. This study was performed on a subset of individuals, as described in Section 2.1. Namely, genome-wide DNA methylation was analyzed in the hippocampus of six individuals who had died by suicide (mean age (year ± SD) 53 ± 4.98; mean PMI interval (h ± SD) 21.58 ± 13.74) and six control subjects (mean age (year ± SD) 52.67 ± 4.27; mean PMI interval (h ± SD) 17.5 ± 3.62). We prepared a list of genes that met the presupposed criteria. We defined the criteria as genes containing at least one differentially methylated cytosine (DMC) with at least a 5% difference in DNA methylation, and with a q-value (*p*-value adjusted for multiple testing) lower than 0.01. Genes of interest that met the proposed criteria were then selected after a narrative literature review and consultation with the research group from the Bradshaw Lab, Faculty of Biotechnology and Drug Development, University of Rijeka, Croatia, which studies protein aggregation. This narrowed the selection down to nine genes of interest (detailed in Table 2) that have been implicated in the literature to be involved in proteostasis and/or neurological or psychiatric disorders. The nine candidate genes were *CRMP1*, *DISC1*, *TSNAX–DISC1*, *MAPT*, *SOD1*, *PRKN*, *GABARAPL1*, *GRIN2A*, and *GRIN2B*. Three reference genes were selected, including *GAPDH* (a commonly used reference gene), *BECN1*, and *DCTN2* (both selected based on our previous validation (publication in preparation, data available upon request)).

### 2.4. RNA Isolation and Gene Expression

RNA was isolated from 30 micrograms of powdered frozen brain tissue, using the phenol–guanidine isothiocyanate-based protocol with TRIzol™ reagent (Thermo Fisher Scientific, Waltham, MA, USA). The concentration and purity of the isolated RNA was evaluated using Synergy H4 (BioTek, Shoreline, WA, USA). Following the manufacturer’s instructions, equal amounts of RNA per subject sample were transcribed to cDNA using the High Capacity cDNA Reverse Transcription Kit (Thermo Fisher Scientific, Waltham, MA, USA) and the GeneAmp™ PCR System 9700 (Applied Biosystems, Waltham, MA, USA).

All qPCR experiments were carried out following MIQE guidelines [47], using TaqMan™ Gene Expression Assay hydrolysis probes, qPCR TaqMan™ Fast Advanced Master Mix (both Applied Biosystems™, Thermo Fisher Scientific, Waltham, MA, USA), and QuantStudio™ 5 (Thermo Fisher Scientific, Waltham, MA, USA), all following the manufacturer’s instructions. Details of reference and candidate genes, including the hydrolysis probe number, are available in Table 2. Hydrolysis probes were first validated on a pooled sample of each study group. All qPCR reactions were run in triplicate, including a no template control.

### 2.5. Statistical Analysis

DNA methylation data was previously analyzed, as described in Kouter et al. (2019) [21]. Using R Studio, R version 4.5.0, genome-wide RRBS DNA methylation data was filtered based on genomic position, percentage difference in DNA methylation per cytosine, and q-value [48].

Using the cycle of quantification data values, geometric averaging was applied, as described by Vandesompele et al. (2002) [49]. Statistical analysis and data visualization were generated using GraphPad Prism version 10.0.0 (GraphPad Software, Boston, MA, USA, www.graphpad.com). The normality of the gene expression and the *GRIN2A/GRIN2B* ratio data distribution were checked using the D’Agostino–Pearson, Anderson–Darling, Shapiro–Wilk, and Kolmogorov–Smirnov tests. A data comparison between the individuals who had died by suicide and the control group subjects was performed using the Student’s *t*-test for normally distributed data, and the Mann–Whitney test for data that did not follow a normal distribution. The considered limit of statistical significance was a *p*-value < 0.05.

## 3. Results

### 3.1. DNA Methylation Reanalysis in the Hippocampus

In the hippocampus, the DNA methylation status of selected candidate genes was further reanalyzed. Detailed information is presented in Table 3. We observed lower levels of DNA methylation in individuals who had died by suicide, with the exception of *CRMP1*, where two of eight DMC were hypermethylated (mean DNA methylation percentage values of 15.64 and 5.64 per DMC, respectively, as detailed in Table 3) in individuals who had died by suicide compared to control group subjects. Candidate genes varied in the number of DMC, from the highest number in *CRMP1* (eight DMC), and the lowest in *PRKN*, *GABARAPL1*, and *GRIN2B* (each gene having one DMC). Looking at the DNA methylation percentage difference between the cases and the controls, the genes with the biggest and smallest percentage difference were *SOD1* (absolute average of 27.62%) and *PRKN* (absolute value of 9.7%).

### 3.2. Gene Expression

Relative gene expression of nine candidate genes was compared between the individuals who had died by suicide and the control group subjects. A comparison was made in two brain regions, in the hippocampus and in Brodmann area 46. For both brain regions, we calculated the ratio of *GRIN2A*/*GRIN2B*.

#### 3.2.1. Gene Expression in the Hippocampus

**Figure 1:**
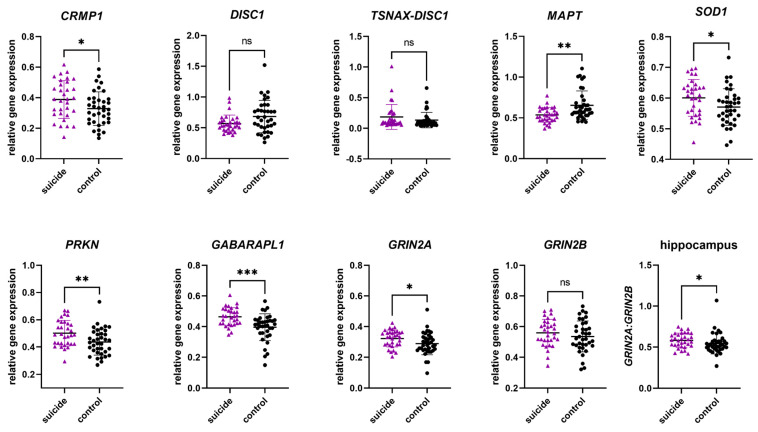
Relative gene expression of candidate genes in the hippocampus. Data are presented in scatter plots with mean and SD, with each point as a measure of relative gene expression per subject. Tests used are Mann–Whitey U test or Student *t*-test. Abbreviations: ns—not statistically significant; *p*-value > 0.05; *—*p*-value ≤ 0.05; **—*p*-value ≤ 0.01; ***—*p*-value ≤ 0.001.

In the hippocampus, we observed significant differences in gene expression between the cases and the controls in six of nine genes. For individuals who had died by suicide, mean gene expression levels were significantly increased in *CRMP1*, *SOD1*, *PRKN*, *GABARAPL1*, and *GRIN2A*, while *MAPT* revealed decreased levels. While not significant, *DISC1* indicated a trend towards decreased mean levels of gene expression in individuals who had died by suicide. No significant difference in levels of *TSNAX–DISC1* and *GRIN2B* were observed. Analyzing the *GRIN2A*/*GRIN2B* ratio, a significant increase can be noticed in individuals who had died by suicide. More details can be found in Table 4 and Figure 1.

#### 3.2.2. Gene Expression in Brodmann Area 46

**Figure 2:**
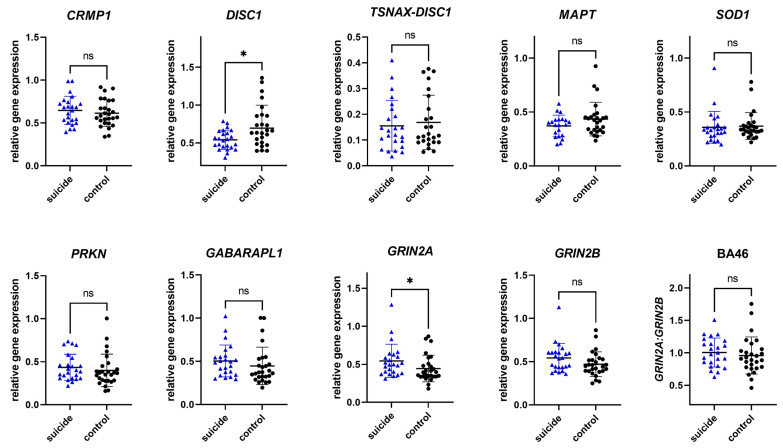
Relative gene expression of candidate genes in Brodmann area 46. Data are presented in scatter plots with mean and SD, with each point as a measure of relative gene expression per subject. Tests used are Mann–Whitey U test or Student *t*-test. Abbreviations: ns—not statistically significant; *p*-value > 0.05; *—*p*-value ≤ 0.05.

To investigate whether the observed changes in gene expression were tissue specific, gene expression was analyzed in an additional brain region, BA46. In BA46, we observed significant differences in gene expression between the cases and the controls in two of nine genes. For individuals who had died by suicide, mean gene expression levels were significantly increased in *GRIN2A*, while *DISC1* revealed decreased levels. While not significant, *GABARAPL1* and *GRIN2B* showed a trend towards increased mean levels of gene expression in individuals who had died by suicide. No significant difference in levels of *CRMP1*, *TSNAX–DISC1, MAPT, SOD1,* and *PRKN* were observed. Analyzing the *GRIN2A*/*GRIN2B* ratio, no significant differences could be observed. More details can be found in Table 5 and Figure 2.

## 4. Discussion

The aim of our study was to investigate the differences in DNA methylation and gene expression of candidate genes that may play a role in protein aggregation between individuals who had died by suicide and a control group. To our knowledge, this is the first study to examine DNA methylation and gene expression of protein aggregation-associated genes in the brains of individuals who had died by suicide. The genes investigated were selected based on the results of a study of DNA methylation status in the hippocampus in a Slovenian sample of individuals who had died by suicide [21]. Based on the results obtained by examining the selected genes, looking at the selected genes that were differentially methylated in individuals who had died by suicide compared to the control group, we identified genes that may be related to protein aggregation. Among these, *DISC1* has long been recognized as a genetic risk factor for schizophrenia and affective disorders [33], and GWAS have linked the loci associated with the *DISC1* interactome to suicidality [34]. *GRIN2B* variants have been associated with treatment-resistant depression [35]. *MAPT* has also been linked to mood dysregulation and cognitive symptoms in psychiatric populations. While transcriptional alterations may indicate dysregulation of the pathways related to proteostasis, they cannot provide direct evidence of aggregate formation.

The qPCR results showed that there were significant differences in gene expression between individuals who had died by suicide and a control group in both brain regions examined: the hippocampus and BA46. We also observed a statistically significant difference in the expression of the mRNA ratio of the NMDAR subunits, *GRIN2A*/*GRIN2B*, but only in the hippocampus.

While the findings of our genome-wide DNA methylation study were exploratory and preliminary, overall, across the selected candidate genes, we observed a decrease in DNA methylation levels in the hippocampus of individuals who had died by suicide. Throughout the selected candidate genes, overall, we observed a decrease in the levels of DNA methylation in the hippocampus of individuals who had died by suicide. While DNA methylation is only one of the factors that influence gene expression, association (but not causality) between decreased levels of DNA methylation in DMCs and increased mean levels of gene expression in the hippocampus can be observed for most of the genes we examined. The exceptions were *DISC1* and *MAPT*, where the mean gene expression appeared lower (although not always significantly) in the hippocampus (*DISC1 p*-value 0.0642 and *MAPT p*-value 0.0020) in individuals who had died by suicide, despite observed hypomethylation of individual sites within the gene.

As we wished to evaluate tissue specificity, we explored gene expression in BA46 as well as in the hippocampus. A similar trend of gene expression levels was observed in BA46, where most genes had increased mean expression levels in individuals who had died by suicide, but the differences between the groups were not significant (with the exception of the *DISC1 p*-value 0.0241 and the *GRIN2A p*-value 0.0232). As in the hippocampus, the genes showing lower mean expression in BA46 were *DISC1* (*p*-value 0.0241) and *MAPT* (*p*-value 0.1691), indicating that such genes might show significance in a larger study.

*CRMP1* encodes one of five homologous cytosolic proteins (CRMP1) with high expression in the central nervous system [50]. The cytosolic phosphorylated protein CRMP1 is part of the semaphorin 3A signaling pathway and is involved in the proper development of the central nervous system and the regulation of neuronal migration [51]. It cooperates with reelin in the regulation of neuronal migration; reelin has previously been associated with neuropsychiatric disorders and neurodegeneration [52]. We observed significantly higher *CRMP1* expression in the hippocampus of individuals who had died by suicide (*p*-value 0.0369). Similar results were reported by Bader et al., who observed an increased expression of *CRMP1* in the blood of individuals with schizophrenia [51]. They also hypothesized that increased mRNA expression in lymphoblast cells is associated with higher protein levels and that CRMP1 has an increased tendency to aggregate when overexpressed [51].

*DISC1* is one of the genes known to be strongly associated with the development of many mental disorders, particularly schizophrenia, and is thus an established genetic risk factor [53]. It encodes the DISC1 scaffold protein, which is involved in neurotransmitter signaling through interactions with a number of other proteins and is involved in numerous processes of development and maturation of the nervous system, such as neurite outgrowth and cerebral cortex development [54]. To date, studies on brain samples of individuals with schizophrenia, MDD, and bipolar disorder have demonstrated the presence of insoluble aggregates of the DISC1 protein. The name DISC1opathy has been proposed for diseases associated with DISC1 aggregation [33]. The putative role of the protein’s own properties in aggregation is supported by the study of Zaharija and Bradshaw, who showed that a structural region of the protein, which is about 30 amino acids long and prone to aggregation, is crucial for aggregation [32]. Of specific interest is the observation that CRMP1 and DISC1 appear to co-aggregate [30]. The results of our study showed a significant difference in *DISC1* expression in BA46 in individuals who had died by suicide (*p*-value 0.0241), while it was not significant in the hippocampus (*p*-value 0.0642). The decreased levels of DNA methylation in *DISC1* have previously been associated with mental disorders. An analysis in the blood of the original t(1;11) translocation family in which the *DISC1* gene was first identified showed 13 differentially methylated sites, four of which were located in *DISC1* [55].

We also investigated the gene expression of the *TSNAX–DISC1* gene, which is formed by read-through transcription between the neighboring *TSNAX* and *DISC1* genes. Intergenic excision leads to the formation of the TSNAX–DISC1 fusion protein, which is thought to be involved in neurological development and signaling pathways. Both TSNAX and TSNAX–DISC1 have been associated with mental disorders in previous studies, particularly schizophrenia and bipolar disorder [56]. The role of *TSNAX–DISC1* in the development of potential aggregation therefore requires further investigation, although it did not show significant differences in these analyses.

*MAPT* is a 16-exon gene that encodes the tau protein, with six possible isoforms in the central nervous system. Depending on the brain region, type, and developmental stage of the neuron, different transcripts are expressed [57]. Tau is mainly found in axons, where it is involved in numerous processes, including stabilization and organization of microtubules, axonal transport, maintenance of the structure and function of the synaptic cleft, and signal transmission within numerous neuronal pathways. It has been known for many years that tau is involved in the development of neurodegenerative disorders, known as tauopathies. In individuals with AD and PD, hypomethylation of *MAPT* leads to increased tau expression, while hypermethylation of *MAPT* leads to decreased tau expression [58]. By contrast, our study showed lower *MAPT* expression in the group of individuals who had died by suicide (significant in the hippocampus, *p*-value 0.0020). This is not in agreement with the DNA methylation results that we observed, indicating the possible involvement of other (epi)genetic regulators.

*SOD1* is a gene that codes for the cytoplasmic enzyme superoxide dismutase 1. The enzyme prevents cell damage caused by ROS by converting harmful superoxide radicals into molecular oxygen and hydrogen peroxide, and provides protection against oxidative stress. Recent studies have suggested that *SOD1* is involved in RNA metabolism. As a central transcription factor, it is involved in regulating the expression of certain repair genes and genes related to ROS regulation [59]. The presence of SOD1 protein aggregates has already been detected in the brains of individuals with ALS. In a study by Gagliardi et al., increased *SOD1* gene expression was found in individuals with sporadic ALS, and the increased expression was restricted to the affected brain regions in which the aggregates were present [60]. In our study, *SOD1* gene expression was significantly higher in the hippocampus of individuals who had died by suicide (*p*-value 0.0425). While it may seem plausible that elevated *SOD1* transcription could connect to increased aggregation tendencies, our results do not provide direct evidence for protein aggregation. Nevertheless, the important role of oxidative stress in the development of many mental disorders should not be ignored—the increased gene expression levels could correspond to an increased need for the enzymatic activity of *SOD1* due to increased oxidative stress [61].

*PRKN* is a common causative gene associated with the development of autosomal recessive juvenile PD. It encodes the cytosolic protein Parkin, which functions as an E3 ubiquitin ligase enzyme and is involved in the removal of dysfunctional and redundant proteins as part of the ubiquitin–proteasome system [62]. In addition, Parkin is also involved in the regulation of mitochondrial homeostasis, as it works with the protein PINK1 to remove and degrade dysfunctional mitochondria in the process of mitochondrial autophagy (mitophagy) [63]. The role of *PRKN* and Parkin in the development of mental disorders and psychiatric symptoms is increasingly emphasized. Mental disorders, particularly anxiety and depression, sometimes appear several years before the development of PD symptoms, and an increased risk of developing depression and anxiety has been observed in relatives of patients with early-onset PD [64]. In our study, *PRKN* gene expression was statistically significantly higher in the hippocampus of individuals who had died by suicide (*p*-value 0.0061). In the study by Wang et al., it was shown in cell cultures and animal models that stress factors in PD reduce the solubility of Parkin and lead to the formation of its aggregates [65]. Considering that oxidative stress and stressors similar to neurogenerative disorders are often present in the pathogenesis of mental disorders and suicidality, the increased expression of *PRKN* in suicidal deaths could be explained in several ways. The increased expression could be a consequence of increased oxidative stress, which occurs in many mental disorders, a response to misfolded proteins, or a response to a lack of functional proteins due to the formation of aggregates [66,67]. Further protein-level investigations will be necessary to determine if the observed transcriptional changes translate to impaired proteostasis.

*GABARAPL1* encodes the GABARAPL1 protein, which belongs to the GABARAP family (GABA type A receptor protein). GABARAPL1 plays an important role in autophagy, autophagosome formation, and mitophagy [68]. It directs labeled protein aggregates to the autophagosome through interactions with cargo adaptor proteins that bind and label ubiquitinated protein aggregates for degradation. *GABARAPL1* is expressed in various tissues. High expression of the gene has been found in the central nervous system, where it is involved in the transmission of nerve signals through interactions with the γ2 subunit of the GABAA receptor. The GABAA receptor is present in the synapses of the major inhibitory pathways in the central nervous system and is involved in the regulation of many brain activities. Disruptions in the GABAA receptor function have been linked to the development of some mental disorders, such as anxiety disorders, schizophrenia, and depression [68,69]. GABARAPL1 has also been linked to neurodegeneration in previous studies, mainly reflected in impaired autophagy, which otherwise serves as a protective mechanism against protein aggregates. It has been hypothesized that a decrease in autophagic activity is one of the possible reasons for the progression of neurodegeneration [68]. This assumption is supported by findings in individuals with PD, where low expression of *GABARAPL1* was detected [70]. By contrast, our study showed a significantly higher expression of *GABARAPL1* in the hippocampus (*p*-value 0.0005) of individuals who had died by suicide compared to the control group. In BA46, such significance was not observed (*p*-value 0.0990). One possible explanation for this observed alteration in the hippocampus is the increased demand for proteins of the autophagy system due to a possible initial phase of aggregate formation, which can be compensated by increased levels of these proteins. However, it is quite possible that a completely different mechanism is involved in the development of mental disorders. In the study by Ye et al., they showed that GABARAPL1 and GABARAP participate in the translocation of the GABAA receptor from the intracellular environment to the membrane [69]. Altered protein expression may therefore affect the translocation of the receptor and alter the presence of the receptor at the postsynaptic membrane, which has been described in previous studies in certain mental disorders [71].

The NMDA receptor is a glutamate receptor in the central nervous system that is involved in synaptic signaling, neurological development, and synaptic plasticity, implicating it in memory formation. Glutamate is the main neurotransmitter with excitatory effects. Excessive stimulation of NMDARs can lead to the excitotoxic death of neurons [72]. The latter is considered one of the pathophysiological mechanisms occurring within neurodegeneration and mental disorders [67,73]. The NMDAR is composed of seven subunits. Variants in the *GRIN2A* gene, which encodes the GluN2A subunit, have identified it as one of the most important genes in the pathophysiology of schizophrenia and other neuropsychiatric disorders [74]. In our study, *GRIN2A* expression was observed to be significantly increased both in the hippocampus (*p*-value 0.0368) and in BA46 (*p*-value 0.0232) in individuals who had died by suicide, which is also consistent with the observed lower *GRIN2A* methylation. *GRIN2B* encodes another subunit of the same NMDAR, GluN2B. In our study, we found no differences in mRNA expression between the suicide victims and the control subjects, which is not consistent with findings in the literature. In a study by Brown et al., they examined the expression of *GRIN2B* in a different brain region, the anterior cingulate cortex, and found significant differences in mRNA expression in individuals with MDD and psychosis compared to controls [75].

The physiological function of NMDAR requires the correct mRNA ratio between the GluN2A and GluN2B subunits [75]. We therefore investigated whether the ratio of gene expression of *GRIN2A* and *GRIN2B* subunits of the N-methyl-D-aspartate receptor (NMDAR) in the hippocampus and BA46 region differs between the studied groups. In both brain regions, we observed that the ratio was higher in suicide deaths, and within the hippocampus this difference was also significant (*p*-value 0.0321). This is not in agreement with the results of the studies by Rahman et al. and Brown et al. The former showed a reduced *GRIN2A*/*GRIN2B* mRNA ratio in the dorsolateral prefrontal cortex of deceased patients with schizophrenia [76]. Similarly, the latter study described a reduced *GRIN2A*/*GRIN2B* mRNA ratio in individuals with MDD and psychosis [75]. One possible reason for the inconsistency in the results is the differences in the study group. Both studies examined individuals with schizophrenia, and the cause of death in all subjects was not suicide. The reduced *GRIN2A*/*GRIN2B* ratio in these studies may therefore be more likely to be associated with schizophrenia or psychosis. Despite contradictory results, we were able to demonstrate changes in the balance of subunit expression and a possible link between altered expression and suicidality.

Our study has some limitations worth mentioning. DNA methylation information was available only for one brain region (hippocampus), for a small subset of subjects (n = 12), and did not contain information regarding the whole genome due to the experimental setup (reduced representation bisulfite sequencing), restricting our study to an exploratory study. Due to national legislation restrictions, limited data on a subject’s health history is available, excluding data on medication status, psychiatric, and cardiovascular comorbidities among participants. As oxidative stress, which is involved in the pathogenesis of cardiovascular disease, also influences DNA methylation patterns [77], this presents an important drawback. However, the selection of sudden death controls is explicitly recommended in brain banking guidelines because it minimizes important confounding factors such as agonal state and tissue integrity [78]. While the observed alterations in suicide cases may reflect brain-specific and suicide-related epigenetic mechanisms, we cannot exclude potential effects of cardiovascular disease-related oxidative stress on DNA methylation.

Due to the nature of the study population, the observed associations between DNA methylation and gene expression are correlational and do not prove causality, and would not all remain significant after correction for multiple testing. Gene expression results were not corrected for multiple testing due to the exploratory nature of this study. Therefore, these findings should be interpreted with caution and confirmed in larger, independent cohorts. Conversely, some of the non-significant but suggestive *p*-values might become significant in larger studies. DNA methylation analysis was performed in a subset of 12 individuals, and gene expression was performed in a larger cohort of 69 individuals, so direct correlations between methylation and expression cannot be confirmed. Consequently, any association between DNA methylation and gene expression should be considered indirect. Furthermore, our study does not provide direct biochemical or histopathological evidence of protein aggregation. Validation in larger cohorts and further protein-based experiments are needed. Despite the aforementioned limitations, this study is important. It opens up and supports a new area of investigation into the possible role of protein aggregation in mental disorders. Because of the tissue specificity of epigenetics and the localization of aggregates in the brain, post-mortem studies (despite their obvious limitations, such as subject heterogeneity) are crucial for understanding psychopathology, especially in a high-risk population, such as in Slovenia. The results suggest that suicide is associated with gene-specific hypomethylation and altered gene expression in stress, plasticity, and synapse-related pathways, particularly in the hippocampus. The detected differences in DNA methylation and gene expression suggest a possible role of proteostasis disorders and protein aggregate formation in psychopathologies, including suicidality, although the effects could be heterogeneous across genes and brain regions.

## Figures and Tables

**Table 1:** Age and PMI of the subjects included in this study. Data are presented with mean ± SD. The Mann–Whitney test was used to determine differences in the age structure and the post-mortem interval between the two groups. The limit of statistical significance was a *p*-value < 0.05.

Subjects	Age (Years)	PMI (Hours)
Individuals who died by suicide (*n* = 32)	41.09 ± 12.63	28.83 ± 14.28
Control group subjects (*n* = 37)	56.51 ± 6.83	26.66 ± 18.35
*p*-value	<0.0001	0.5904

PMI: *post-mortem* interval, *n*: number of subjects.

**Table 2:** List of reference and candidate genes whose gene expression was examined by qPCR.

Function	Gene	Assay ID
reference gene	*GAPDH*	Hs02758991_g1
reference gene	*BECN1*	Hs01007018_m1
reference gene	*DCTN2*	Hs00197379_m1
candidate gene	*CRMP1*	Hs00609716_m1
candidate gene	*DISC1*	Hs07287401_m1
candidate gene	*TSNAX–DISC1*	Hs03826399_s1
candidate gene	*MAPT*	Hs00902194_m1
candidate gene	*SOD1*	Hs00533490_m1
candidate gene	*PRKN*	Hs01038323_m1
candidate gene	*GABARAPL1*	Hs00740588_mH
candidate gene	*GRIN2A*	Hs00168219_m1
candidate gene	*GRIN2B*	Hs01002012_m1

**Table 3:** A list of differentially methylated cytosines located within genes of interest. Cutoff criteria were at least 5% difference in DNA methylation between the case and the control group subjects, and a q-value of under 0.01. DNA methylation values are presented as mean differences between the two studied groups, with negative values representing hypomethylation in the group of individuals who had died by suicide.

Gene	Position (hg19)	Gene Location	mDNA % Difference	q-Value
*CRMP1*	chr4:5865655	intron	−16.20	2.25 × 10^−3^
	chr4:5867801	intron	−29.38	1.97 × 10^−11^
	chr4:5872659	intron	−12.38	1.58 × 10^−4^
	chr4:5892328	promoter	−7.81	6.30 × 10^−4^
	chr4:5893333	1 to 5 kb	15.64	2.03 × 10^−3^
	chr4:5893366	1 to 5 kb	−30.80	1.40 × 10^−10^
	chr4:5894303	1 to 5 kb	5.64	2.31 × 10^−5^
	chr4:5899566	1 to 5 kb	−14.31	4.63 × 10^−3^
*DISC1*	chr1:231826172	intron	−18.96	2.62 × 10^−3^
	chr1:232073343	intron	−20.61	2.91 × 10^−5^
	chr1:232073364	intron	−24.51	1.46 × 10^−7^
	chr1:232073393	intron	−32.39	4.30 × 10^−5^
*TSNAX–DISC1*	chr1:231663797	promoter	−9.17	2.81 × 10^−3^
	chr1:231826172	intron	−18.96	2.62 × 10^−3^
	chr1:232073343	intron	−20.61	2.91 × 10^−5^
	chr1:232073364	intron	−24.51	1.46 × 10^−7^
	chr1:232073393	intron	−32.39	4.30 × 10^−5^
*MAPT*	chr17:43974616	intron	−10.79	1.32 × 10^−3^
	chr17:43974622	intron	−15.80	2.94 × 10^−6^
	chr17:43974635	intron	−11.40	5.34 × 10^−4^
	chr17:44101476	exon	−18.77	2.25 × 10^−4^
	chr17:44101486	exon	−25.83	3.78 × 10^−8^
	chr17:44101491	exon	−22.69	3.19 × 10^−6^
*SOD1*	chr21:33031523	promoter	−24.27	6.00 × 10^−8^
	chr21:33031539	promoter	−30.97	2.15 × 10^−12^
*PRKN*	chr1:89166724	intron	−9.70	4.27 × 10^−3^
*GABARAPL1*	chr12:10368728	intron	−26.63	7.32 × 10^−5^
*GRIN2A*	chr16:9970416	intron	−21.76	1.13 × 10^−6^
	chr16:10001979	intron	−32.04	1.36 × 10^−5^
	chr16:10070665	intron	−12.92	4.80 × 10^−3^
	chr16:10277015	promoter	−5.26	8.29 × 10^−4^
*GRIN2B*	chr12:13989920	intron	−16.79	9.57 × 10^−3^
	chr12:13994346	intron	−7.21	2.49 × 10^−3^
	chr12:14108596	intron	−54.37	3.20 × 10^−18^

mDNA—DNA methylation.

**Table 4:** Gene expression in the hippocampus.

Gene	*p*-Value	Mann–Whitney or Student *T*-Test Statistics
*CRMP1*	0.0369	t = 2.129, df = 67, 95% CI [0.003–0.028]
*DISC1*	0.0642	U = 425, mean S = 0.5419, mean C = 0.6515
*TSNAX–DISC1*	0.2376	U = 493, mean S = 0.1031, mean C = 0.09710
*MAPT*	0.0020	U = 339, mean S = 0.5323, mean C = 0.6006
*SOD1*	0.0425	t = 2.069, df = 67, % CI [−0.05864–0.001046]
*PRKN*	0.0061	t = 2.834, df = 67, % CI [−0.1108–0.01921]
*GABARAPL1*	0.0005	U = 296, mean S = 0.4679, mean C = 0.4127
*GRIN2A*	0.0368	t = 2.131, df = 67, % CI [−0.06504–0.002128]
*GRIN2B*	0.3067	t = 1.030, df = 67, 95% CI [−0.06969–0.02225]
*GRIN2A/GRIN2B*	0.0321	U = 414, mean S = 0.5827, mean C = 0.5220

U—Mann–Whitney U value; S—suicide, C—control; % CI—95% confidence interval; df—degrees of freedom.

**Table 5:** Gene expression in Brodmann area 46.

Gene	*p*-Value	Mann–Whitney or Student *T*-Test Statistics
*CRMP1*	0.4662	t = 0.7344, df = 49, % CI [−0.1233–0.05729]
*DISC1*	0.0241	t = 2.328, df = 49, % CI [0.02117–0.2882]
*TSNAX–DISC1*	0.7738	U = 285, mean S = 0.1286, mean C = 0.1173
*MAPT*	0.1691	U = 208, mean S = 0.3823, mean C = 0.4231
*SOD1*	0.4942	U = 287, mean S = 0.3343, mean C = 0.3374
*PRKN*	0.2482	U = 262, mean S = 0.3947, mean C = 0.3682
*GABARAPL1*	0.0990	U = 236, mean S = 0.4927, mean C = 0.3725
*GRIN2A*	0.0232	U = 204, mean S = 0.4979, mean C = 0.3946
*GRIN2B*	0.0914	U = 234, mean S = 0.5405, mean C = 0.4495
*GRIN2A/GRIN2B*	0.3436	U = 273, mean S = 0.9923, mean C = 0.9107

U—Mann–Whitney U value; S—suicide, C—control; % CI—95% confidence interval; df—degrees of freedom.

## Data Availability

The data presented in this study are available upon request from the corresponding author due to privacy and ethical reasons of post-mortem data.

## Supplementary Materials

The following supporting information can be downloaded at: https://www.mdpi.com/article/10.3390/genes16121467/s1, Table S1: Detailed subject data.

## Abbreviations

The following abbreviations are used in this manuscript:

AD	Alzheimer’s disease
ALS	amyotrophic lateral sclerosis
*DISC1*	disrupted-in-schizophrenia 1
DMC	differentially methylated cytosine
*DTNBP1*	dystrobrevin-binding protein 1
FTD	frontotemporal dementia
GWAS	genome-wide association studies
HPA	hypothalamic–pituitary–adrenal
MDD	major depressive disorder
mDNA	DNA methylation
NMDAR	N-methyl-D-aspartate receptor
*NPAS3*	neuronal PAS domain protein 3
PD	Parkinson’s disease
PMI	*Post-mortem* interval
*TRIOBP*	TRIO and F-actin binding protein
