# Supplementary material for: Epigenetic and Transcriptomic Alterations of Protein Aggregation-Linked Genes in Suicide: A Pilot Study
Source: Genes (Basel). 2025 Dec 8;16(12):1467. doi: 10.3390/genes16121467 (PMC12733106; doi:10.3390/genes16121467)
Supplement: Supplementary file 1 [file genes-16-01467-s001.zip › genes-3924090-supplementary.pdf]

Supplementary

Epigenetic and transcriptomic alterations of protein aggregation-linked genes in suicide

Taja Bedene [1]†, Julija Šmon [2]†, Alja Videtič Paska [2], Tomaž Zupanc [3] and Katarina Kouter [4,*]

[1] Faculty of Medicine, University of Ljubljana, 1000 Ljubljana, Slovenia; taja.bedene10@gmail.com
[2] Institute of biochemistry and molecular genetics, Faculty of Medicine, University of Ljubljana, 1000 Ljubljana, Slovenia; julija.smon@mf.uni-lj.si; alja.videtic@mf.uni-lj.si
[3] Institute of forensic medicine, Faculty of Medicine, University of Ljubljana, 1000 Ljubljana, Slovenia; tomaz.zupanc@mf.uni-lj.si
[4] Institute of microbiology and immunology, Faculty of Medicine, University of Ljubljana, 1000 Ljubljana, Slovenia; katarina.kouter@mf.uni-lj.si
† These authors contributed equally to this work

* Correspondence: katarina.kouter@mf.uni-lj.si

Subject information

Table S1: Detailed subject data.

Table contains data of subject status (suicide victims are coded with S, control group is coded with C), age, post mortem interval (PMI), active prescriptions for the duration of five years before death and toxicology results from the autopsy report.

Subject	Age (years)	PMI (h)	Active prescriptions	Toxicology
C10	52	17	-	negative
C11	47	58	-	negative
C12	60	54	-	negative
C13	59	24	-	negative
C14	64	22	-	-
C15	59	34	-	negative
C16	61	18	-	negative
C17	50	15	-	negative
C18	59	20	-	negative
C19	53	25	-	negative
C2	54	19	-	negative
C20	33	16	-	negative
C21	60	13	-	positive for ethanol
C22	49	59	-	positive for antiepileptics and antipsychotics
C23	60	106.5	-	positive for ethanol

C24	64	17	-	negative
C25	63	32	-	positive for ethanol
C26	63	23	-	positive for ethanol
C27	57	19	-	negative
C28	63	20	-	positive for ethanol
C29	53	16	-	positive for anxiolytics
C3	47	12	-	negative
C30	49	13	-	negative
C31	57	15	-	negative
C32	58	23	-	negative
C34	61	7	-	negative
C36	65	29	-	negative
C37	64	25	-	negative
C39	57	19	-	negative
C4	55	17	-	negative
C40	65	27	-	negative
C41	59	17	-	negative
C5	60	48	-	negative
C6	50	20	-	positive for ethanol
C7	45	37	-	negative
C8	59	22	-	negative
C9	57	28	-	negative
S1	38	12	none	negative
S11	23	39	none	positive for ethanol and cocaine
S12	35	51.5	none	positive for ethanol
S13	32	61	none	positive for ethanol
S14	21	30	none	positive for ethanol
S15	48	40	none	positive for antidepressants
S16	54	23	none	negative
S17	39	33	none	positive for ethanol
S18	32	32.5	none	positive for ethanol
S19	46	36	none	positive for ethanol

S2	60	22.5	none	negative
S21	18	36	none	positive for ethanol
S22	30	13	none	positive for ethanol
S23[4]	60	23	antipsychotics antidepressants	positive for antidepressants and antipsychotics
S25	33	16.5	none	negative
S26	42	17	-	positive for ethanol
S27	33	21	-	negative
S28	64	20	-	negative
S29	29	34	-	negative
S3	39	28	none	negative
S30	22	46	-	negative
S31	45	38	-	negative
S32	31	7	-	negative
S33	50	63	-	negative
S36	36	26	-	positive for anxiolytics
S37	37	24	-	negative
S4[1]	46	9.5	antipsychotics	positive for antidepressants and antipsychotics
S5	54	36.5	none	positive for ethanol
S6[2]	51	15	hypnotics and sedatives antipsychotics	positive for ethanol positive for antidepressants and anxiolytics
S7[3]	59	15	hypnotics and sedatives antipsychotics	positive for antidepressants
S8	58	41.5	none	positive for ethanol
S9	50	12	none	positive for ethanol

1Suicide victim S4 diagnosed with schizophrenia, died on the day of being released from hospital.
2 Suicide victim S6 diagnosed with schizophrenia.
3 Suicide victim S7 diagnosed with major depressive disorder, died on the day of being released from hospital.
4 Suicide victim S23 diagnosed with adjustment disorder, died on the fourth day of being released from hospital.
– no data available due to national data restrictions
